# Speech analytics across the schizophrenia spectrum disorders: multimodal natural language processing and machine learning modelling in a Chinese-speaking population

**DOI:** 10.3389/fpsyt.2025.1725859

**Published:** 2026-01-06

**Authors:** Jiaqi Liu, Sumiao Zhou, Guangxing Deng, Meng Ji, Xufei Zhu, Xue He, Qijie Kuang, Shenglin She

**Affiliations:** 1 Department of Psychiatry, The Affiliated Brain Hospital, Guangzhou Medical University, Guangzhou, China; 2 Guangdong Engineering Technology Research Center for Translational Medicine of Mental Disorders, Guangzhou Medical University, Guangzhou, China

**Keywords:** formal thought disorder, machine learning, natural language processing, schizophrenia spectrum disorders, speech

## Abstract

**Background:**

Formal thought disorder (FTD) is a core symptom of schizophrenia spectrum disorders (SSDs). As a key representational dimension of FTD, speech features have been shown in previous studies to hold potential as diagnostic biomarkers for SSD. However, relevant research remains limited, and such speech features have not yet been applied clinically for SSD diagnosis.

**Objective:**

The aim of this research is to establish a Chinese speech database for multidimensional analysis of speech characteristics, quantify these high-dimensional linguistic features using natural language processing (NLP), and ultimately develop objective biomarkers for diagnosing and assessing the severity of SSD.

**Methods:**

This will be a single-center, prospective, observational study. In accordance with the DSM-5 criteria, a total of 300 inpatients or outpatients meeting the diagnostic criteria for SSD are planned to be included. Healthy controls with no history of intellectual disability will subsequently be matched. Each participant will undergo a 1-to-2-hour task-guided interview conducted by a psychiatrist, which includes an app-based assessment of the PANSS(Positive and Negative Syndrome Scale), short passage reading, an animal fluency test, a pseudosentence reading task, a symptom severity rating task, an inner-world expression task, and a picture description task. All the interviews will be audio-recorded. After the interview, clinical rating scales will assess psychiatric symptom severity, social functioning, and thought-language disorders. During the study, at an interval of 2 weeks.

**Discussion:**

By multidimensionally quantifying these speech characteristics and integrating machine learning, this study aims to screen highly discriminative speech feature combinations specific to SSD, thereby providing technical and theoretical support for the precise diagnosis and personalized intervention of SSD. These findings will deepen psychiatrists’ understanding of the linguistic pathological mechanisms underlying SSD and promote the development of diagnostic tools and intervention protocols based on novel biomarkers.

## Introduction

Since 1990, the prevalence, incidence, and burden of schizophrenia have continued to increase (1). The global lifetime prevalence is approximately 1% (2), imposing a substantial burden on individuals, families, and society (3). Schizophrenia spectrum disorders (SSDs) are characterized by positive symptoms such as hallucinations, delusions and formal thought disorder (FTD); negative symptoms such as affective flattening, avolition and others; and cognitive dysfunction. Among these, formal thought disorder is among the most frequently occurring symptoms in SSD patients (4). FTD refers to disorganized and incoherent thought processes. Surveys of patients with schizophrenia report that its prevalence ranges from 25% to 75% (5).

FTD is primarily expressed through speech characteristics, manifesting as incoherent and disorganized speech (6, 7). Studies have shown that the nodes of the semantic networks in speech from FTD individuals with schizophrenia are more scattered than those from healthy participants (8). In terms of prosody, flat intonation occurs frequently among individuals with schizophrenia (9). With increasing FTD, the syntax becomes simpler (10). Speech characteristics correlate with both positive and negative symptoms in individuals with SSD. For example, conceptual disorganization (a positive symptom) involves disrupted thought coherence, such as tangential or verbose speech. In contrast, negative symptoms manifest as verbal poverty (e.g., brief, empty responses) and impaired emotional communication, which is characterized by rigid, unnatural conversations and potentially mechanical replies. Previous studies have shown that mathematical models built from acoustic parameters, including loudness, formant bandwidth, and amplitude, in individuals with schizophrenia correlate with PANSS negative symptom scores (11). In fact, FTD exists in the early stages of SSD and may be a biomarker of illness severity in the early stages of psychosis (12). A study using an FTD assessment method based on word connectivity reported 85% accuracy in distinguishing individuals with schizophrenia from healthy controls (13). Furthermore, studies have shown that speech features can serve as quantitative indicators with high accuracy for diagnosing SSD and are highly specific to psychosis (11). These studies indicate that speech features hold considerable potential in the development of digital SSD phenotypes. However, individuals with SSD exhibit significant clinical heterogeneity, as their speech manifestations show not only individual variability but also dynamic changes over time, making consistent analysis challenging. Traditional language analysis methods, which struggle to account for such variability, lack objectivity and reproducibility.

With the development of artificial intelligence (AI) and natural language processing (NLP) methods based on machine learning (ML), existing computational tools can extract linguistic features more accurately, including speech content and physical characteristics, and apply them to disease classification and functional evaluations (14). In recent years, studies using ML-based NLP techniques to distinguish psychiatric disorders, including schizophrenia (SZ) and affective disorders, have become increasingly common (15–17). Corcoran et al. validated ML classifiers based on NLP methods using part-of-speech tagging analysis (for measuring syntactic complexity) and latent semantic analysis (for measuring semantic coherence) to predict psychosis onset (18). Nonetheless, current related studies still have issues of insufficient comparability and reproducibility. The application of automated NLP analysis of speech features in diagnosis and treatment across neuropsychiatry remains in its infancy.

In recent years, several exploratory studies on Asian samples have laid an important foundation for cross-linguistic research on SSD (19, 20). Meanwhile, relevant research in the Chinese context still has room for further expansion and refinement: in terms of sample size, the sample sizes of existing studies are mostly concentrated around 40–50 cases, and large-scale, cross-regional datasets have not yet been established; in terms of research methods, previous studies have mostly adopted a single task paradigm (e.g., picture description task), with relatively limited types of linguistic texts analyzed, making it difficult to comprehensively and systematically capture the multidimensional manifestations of speech features in patients with SSD.

## Methods

### Dataset creation

In this section, we describe the methods for creating large Chinese datasets of adult SSD patients and healthy controls. We plan to make the datasets openly available. After completing the ethical approval modification process, they will be available to researchers with legitimate needs in the field of mental illness language science.

### Participants

This study is a multicenter, prospective, observational study. Subject recruitment began in 2025 and remains ongoing. Participants were recruited from psychiatric outpatient clinics or inpatient departments of psychiatric hospitals and general hospitals in South China. The inclusion criteria for patients with SSD were as follows: (i) outpatients or inpatients meeting the Diagnostic and Statistical Manual of Mental Disorders-Five Edition (DSM-5) diagnostic criteria (including acute and transient psychotic disorders, schizophrenia, schizoaffective disorders, delusional disorders, etc.); (ii) right-handed, aged 18 to 59, with an educational level ≥ 9 years; (iii) a Positive and Negative Syndrome Scale (PANSS) score ≥ 60; and (iv) speaks Mandarin fluently. The exclusion criteria included nervous system disease, intellectual disability, hearing loss, alcohol or drug abuse, treatment with electroconvulsive therapy within the past six months, and inability to cooperate with the study because of physical illness or acute mental status. All patients with SSD received antipsychotic medication during the study, and their condition was stable.

Healthy volunteers will be healthy individuals with no history of psychiatric disorders who volunteered via the research team’s website or recruitment advertisements and aged ≥ 18 years. Written informed consent for study participation, data storage, and the use of de-identified data for academic research-related public purposes will be obtained from all healthy volunteers; for patients, it will be signed by their legal guardians. Participants may withdraw at any time. All procedures involving human subjects/patients were approved by the Medical Ethics Committee of The Affiliated Brain Hospital, Guangzhou Medical University.

### Data collection

In this study, demographic characteristics and language data (voice and text information) were collected. We assigned a study number to each participant and managed the data accordingly. The clinical assessment results were linked to the study number and stored separately from identifiers such as name and address. Voice data, which might contain identifiers, was stored strictly on a password-locked server. Such identifiers were removed from the public dataset.

### Demographic characteristics

Sex, age, educational background, diagnosis, disease course, age of onset, treatment medications, and complications will be collected from medical records. We will also collect as much information as possible about contact details, address, outpatient or inpatient status, occupation, employment status, and marital status. If some information is missing, participants will be contacted via phone or email to provide it.

### Conversation data

**Figure 1:**
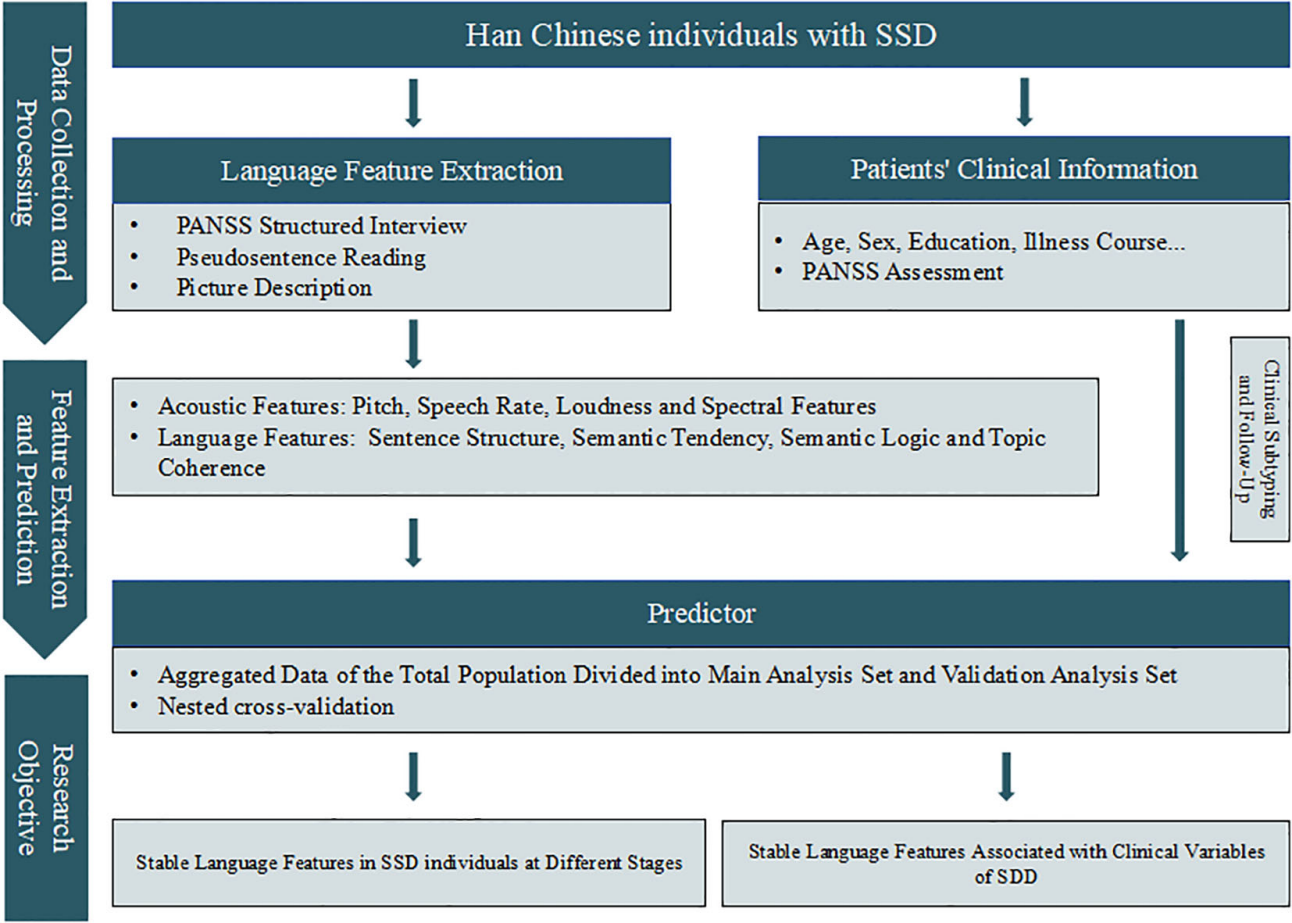
SSD speech feature analysis flowchart. SSD, schizophrenia spectrum disorders; PANSS, Positive and Negative Syndrome Scale.

All the subjects underwent speech data collection in a quiet assessment room. Recordings were made using a ZOOM H1N recorder (with a bit rate of 24 bits and a sampling rate of 96 kHz). During the collection process, the distance between the subjects’ heads and the microphone was maintained between 0.5 and 1.0 m. Before the tasks began, assessors instructed the subjects to avoid noise-generating activities as much as possible. For the analysis of linguistic features in this study, data preprocessing is first conducted, including steps such as denoising and standardization, to ensure data quality. Subsequently, the acquired speech data need to be converted into text using speech recognition technology, followed by manual correction to guarantee text accuracy. Afterwards, the data are processed using automatic language calculation methods. The tasks are designed in ascending order of cognitive load: First, the SCI-PANSS is used to establish the clinical background, followed by a series of evaluation tasks, specifically including the following (**Figure 1**).

### PANSS assessment

All participants will complete the PANSS assessment to quantify their psychiatric symptoms over the past week and establish a basic clinical profile. The assessment will be conducted using a researcher-developed app integrated with NeuroVoice software for structured interviews. Researchers will remain silent throughout, providing only brief pretask instructions; the entire process will be guided and managed by the app. Equipped with an AI voice generation system, the app will automatically ask standardized, neutral questions one by one. Participants will respond in natural language, enabling human–machine voice interaction to avoid subjective researcher interference and ensure a standardized environment. The app will also automatically record response timestamps and voice features (e.g., duration, speech rate, intonation). After the completion of the APP-based questionnaire, the assessor conducts open-ended follow-up questions based on the participants’ responses to further verify symptoms and ensure the reliability of the scale assessment. Notably, no speech data is collected during the open-ended questioning phase. Currently, this software is in Version 1.0, and subsequent iterations will be carried out to launch a public multilingual version for researchers worldwide.

### Short text reading task

Participants will read the classic short text *The North Wind and the Sun* to assess their basic language abilities, speech fluency, and pronunciation accuracy.

### Animal fluency task

Participants will name as many different animals as possible within 1 minute. This task assesses language fluency, thought organization, and lexical fluency.

### Pseudosentence reading task

Participants will read aloud a set of pseudosentences generated from publicly available textual materials. These pseudosentences all follow the grammatical structure of “subject + predicate + object,” with each containing 12 Chinese characters, including 3 key words. For example, the English translation of a Chinese nonsense sentence “一个男声可能复习两个课本” is “A male voice may review two textbooks.” The keywords underlined are subject, predicate and object. The pseudosentences are grammatically correct but lack actual semantic content, and the number of Chinese syllables is balanced across sentences. Prior to formal use, all pseudosentences were checked by two researchers to verify their grammatical correctness and absence of practical semantic meaning. This task enables the assessment of participants’ language fluency and expressive ability under conditions free from actual semantic load.

### Psychopathological symptoms task

The participants will read text about hallucination and delusion experiences. The text, designed by researchers, is based on common hallucinations and delusions in schizophrenia patients, such as being watched, hearing voices or seeing unreal images.

To create the text scientifically, this study collected self-reported data from 30 schizophrenia patients using semistructured interviews, covering details of their daily hallucinations, delusions and mood swings. All the participants signed written consent forms. Through qualitative analysis, these symptoms were thematically classified, identifying common symptoms such as being watched, controlled or disturbed by voices. The text was developed on the basis of these findings for the reading task.

### Expression of inner thoughts

The participants expressed their reflections and feelings about the previous hallucination and delusion text. This assesses their inner responses to hallucinations and delusions, emotional states, and linguistic expressive abilities at both the emotional and the cognitive levels.

### Picture description task

The participants are required to describe three types of emotional expressions on Chinese facial expressions: positive, neutral, and negative. This task assesses their emotional cognitive processing and linguistic ability in emotional expression.

In this study, linguistic information during disease progression and the correlation between disease severity and linguistic information are highly important. Therefore, participants will be followed up 2 weeks after the baseline assessment, and follow-up data collection will follow the same procedures as the baseline assessment.

### Clinical assessment

After completing the voice tasks, all patients will undergo a systematic clinical evaluation, including assessments of psychiatric symptoms, social functioning, and thought-language disorders. Senior psychiatrists will use the PANSS to evaluate the psychiatric symptoms of patients with SSD over the past week. This assessment will be based exclusively on the PANSS results from the voice tasks, without reinterview. Social functioning will be measured using the Personal and Social Performance Scale (PSP), where lower scores indicate more severe impairment in social functioning. Thought and language disorders will be assessed using the Thought, Language and Communication Scale (TLC) (21). Each symptom is scored from grade 0 (absent) to grade 4 (severe), with the total score reflecting the overall level of thought and communication disorders.

Assessments will be conducted by psychiatrists who have received standardized training, with the assessors remaining blind to the voice data. Prior to the initiation of the assessments, interrater reliability tests will be conducted for all the scale scores (Kappa value > 0.8) to ensure the reliability and scientific validity of the data. Moreover, the assessors will remain blinded to the results of the voice analysis to reduce subjective bias during the assessment process.

### Data processing and annotation

The acquired voice data will be converted into text information using speech recognition technology, with manual correction performed. It will also be annotated, including pauses, repetitions, word types, word counts, erroneous statements, and so forth. When converting voice data into text information, personal identifiers (e.g., names) will be masked. On the basis of the text data obtained above, we will employ natural language processing (NLP) techniques for morphological and syntactic analyses. Specifically, we will use an independently developed Chinese word classifier (22, 23) to extract and calculate various variables. These analyses will include the frequency of each part of speech, vocabulary (negative and positive words), syntactic complexity, speech length, frequency of first-person usage, and use of pronouns, among others. We will use NLP techniques to represent text data as n-grams and word embeddings obtained using pretrained models (e.g., BERT).

In this study, 12 audio metrics will be extracted from the recorded audio data to utilize the physical properties of the audio data in the machine learning (ML) model, including duration (s), mean fundamental frequency (F0) (Hz), standard deviation of F0 (Hz), minimum F0 (Hz), maximum F0 (Hz), harmonics-to-noise ratio (HNR) (dB), jitter (%), shimmer (%), intensity (dB), root mean square (RMS) amplitude (dB), spectral centroid (Hz), and spectral spread (Hz). These audio metrics will be extracted using the Parselmouth package (Praat in Python).

Language features specific to the characteristics of certain mental illnesses will be selected and statistically compared between different groups (e.g., patients vs. healthy subjects and Subgroups of schizophrenia).

### Machine learning

This study will train machine learning models to perform the following tasks: (i) to predict whether subjects have SSD; (ii) to predict disease severity on the basis of scores from scales such as the Positive and Negative Syndrome Scale (PANSS), Personal and Social Performance Scale (PSP), and Thought, Language and Communication Scale (TLC); (iii) to predict changes in a subject’s condition with respect to a previously recorded state if the subject has undergone a prior assessment; and (iv) to predict scores for each item of clinical rating scales, where these items reflect different dimensions of the disease, such as positive symptoms, negative symptoms, and symptoms of disorganization. The model training data will include voice features from voice data, linguistic information from transcribed text data, and demographic data.

Data will be processed, and feature engineering will be performed using natural language processing (NLP) techniques to extract disease features. Machine learning models such as decision trees, support vector machines (SVMs), and deep learning architectures will be employed, and model performance will be evaluated using leave-one-subject-out cross-validation. The feature importance estimation methods will be used to determine the relative importance of each feature, and the prediction results will be assessed using the mean absolute error, coefficient of determination, and correlation coefficient. The extracted features will be validated for their ability to (i) distinguish between normal and disease states, (ii) identify different disease types, and (3) reflect temporal changes in disease severity.

### Statistical analysis

The demographic analyses will be performed using SPSS 24.0. On the basis of different observation indicators and data types, we will describe count data as frequencies (percentages) and measurement data as the mean ± standard deviation. To compare measurement data, we will adopt the independent samples t test; for count data, we will use the chi-square test. We will apply Pearson correlation analysis to explore the relationships between speech features and psychiatric symptoms in schizophrenia patients, while controlling for demographic variables such as age, sex, and educational level. For all the statistical tests above, a P value < 0.05 will be considered to indicate statistical significance.

### Sample size

The sample size was estimated using G-Power 3.1 software. With a two-tailed test set at α = 0.05, a power of 0.95, and an expected effect size of 0.50, 270 samples were required for each group. Considering potential sample loss due to factors such as refusal to participate or poor cooperation in the actual survey, the total sample size was increased by 10%. After adjustment, the sample size was 270 × (1 + 0.10) =297. In summary, approximately 300 patients with SSD need to be enrolled in this study.

## Discussion

This study aims to establish a speech database and diagnostic model for SSD in the Han population by combining diverse speech data collection methods with NLP and machine learning technologies. As of July 2025, we have collected speech data from >100 individuals diagnosed with SSD whose speech data have been initially integrated into a dataset. This study further aims to create a large open Chinese speech dataset, which will provide innovative and objective biomarkers for the diagnosis and treatment of SSD.

Speech dysfunction in SSD patients is closely linked to thought disorders and represents an important clinical feature (24). Compared with clinical reports, the improved accuracy of speech analysis provides more precise symptom monitoring (25). A large body of research indicates that individuals with SSD exhibit significant and systematic abnormalities in speech characteristics during verbal expression (26–28). However, these results are inconsistent. These studies typically use a single speech feature assessment and task paradigm, limiting the types and depth of speech analysis (20, 28, 29).

Our study will use a multitask comprehensive assessment method to evaluate multidimensional functions, including language fluency, grammatical comprehension, language structure, and emotional expression. Data collection will adopt a stepwise task paradigm (e.g., PANSS assessment→short text reading→picture description), covering these dimensions plus repetition of pathological symptoms. Compared with a single task, this approach is more comprehensive and yields a richer dataset. We will use a self-developed app for standardized assessment of PANSS, ensuring the standardization of data collection and providing effective support for multidimensional speech data analysis. Furthermore, this study introduces a novel pseudosentence reading task. Previous speech research in schizophrenia has largely employed picture-description or semispontaneous conversation paradigms, which are susceptible to interference by patients’ cultural backgrounds and cognitive abilities, thereby obscuring their core linguistic profiles. By eliminating semantic content, pseudosentences reduce the semantic processing load and circumvent language-specific constraints, enabling direct examination of formal linguistic features such as Mandarin tone accuracy, pause distribution, and articulator motor coordination (30, 31). Our research will contribute to a comprehensive analysis of speech characteristics in patients with schizophrenia and will improve the accuracy of early diagnosis.

The further development of NLP and ML has significantly enhanced the potential value of linguistic biomarkers. These technologies allow for the rapid and objective measurement of language features (32). Current studies still suffer from insufficient comparability and reproducibility. Many of them are based on small samples, with inadequate cross-validation of the classifiers (33). To address these limitations, our research intends to apply ML techniques with higher classification efficiency to multidimensional speech feature data, screen and validate SSD-specific speech marker combinations with high discriminative power from high-dimensional speech features and construct a high-performance auxiliary diagnostic prediction model for SSD. In subsequent work, we plan to conduct cross-modal analyses using machine learning methods by combining speech features, brain function networks, and abnormal neuroelectrophysiological patterns.

With the help of NLP methods, speech analysis can enable objective quantification of speech abnormalities in SSD. However, most previous SSD speech studies have focused on Western populations. Additionally, several factors, such as sociodemographic factors, clinical heterogeneity, and cross-linguistic variation, can interfere with SSD speech features, particularly in terms of coherence measures (34). Linguistic and cultural differences can exert crucial effects on some quantitative speech markers measured in the subjects (35, 36). This may lead to biases in computational evaluations such as semantic coherence. A study on a global corpus revealed that Chinese and German patients with schizophrenia showed lower coherence compared with controls across several measures. In contrast, compared with controls, Danish patients showed a mixed pattern: higher semantic coherence across multiple measures but lower coherence at the lexical and sentential levels (19). Such differences may be related to the linguistic characteristics of Chinese, which relies on “tone to distinguish meaning,” and Danish, which uses “stress to distinguish meaning.” Additionally, different preprocessing options (e.g., transcript length normalization and the omission of fillers/punctuation marks) can affect various coherence measurements (34). Therefore, there is an urgent need to conduct systematic research on the Han population to explore their unique linguistic manifestations and underlying biological mechanisms. Our study will construct a multi-dimensional speech database for the Han population, filling the research gap concerning the “scarcity of Chinese-language data”.

This study has several limitations. First, although an attempt was made to increase the representativeness of the sample, it failed to cover Han populations from every province, autonomous region, and municipality directly under the Central Government in China. Thus, the initial sample still lacks sufficient representativeness, and we plan to conduct data collection in multiple regions across China in subsequent work. Second, our study included only adult patients with SSD, making it impossible to infer the speech and language characteristics of patients with adolescent-onset or late-onset SSD or to analyze the correlation between age and symptom progression. We aim to expand the sample to include children and adolescents under 18 years old in future studies. Additionally, the unique linguistic features of Chinese individuals increase the difficulty in analyzing the speech characteristics of SSD patients. For instance, Chinese communication commonly involves expressions such as “subject omission” and “inversion,” along with distinct regional diversity in “dialects and cultures,” which may complicate text annotation and analysis.

Despite these challenges, this research will enable us to investigate the language of psychiatric disorders statistically and comprehensively. It will provide psychiatrists with a new interpretation of language.
